# Review of volatile organic compound (VOC) emissions from desktop 3D printers and associated health implications

**DOI:** 10.1038/s41370-025-00778-y

**Published:** 2025-05-08

**Authors:** Danielle A. Baguley, Gareth S. Evans, Delphine Bard, Paul S. Monks, Rebecca L. Cordell

**Affiliations:** 1 University of Leicester, University Rd, Leicester, LE1 7RH UK; 2 University of Northumbria, College Street, Newcastle upon Tyne, NE1 8ST UK; 3 Health and Safety Executive (HSE), Science Division, Harpur Hill, Buxton, SK17 9JN UK

**Keywords:** 3D-printing, Volatile organic compounds, Indoor air quality, Resin, Fused deposition modelling Vat photopolymerization

## Abstract

**Background:**

Three-dimensional (3D) printing is a technique by which materials are continually added in layers to form structures. The technique has grown in popularity over the past decade and affordable desktop 3D printers are now widely used in schools, universities, businesses, and hospitals.

**Objective:**

Understanding the types of chemical emissions from these 3D printers and their potential health effects is essential to safely use this technology.

**Methods:**

A scoping literature review on volatile organic compound (VOC) emissions from resin-bed and filament 3D printers has been conducted. Most of the published research has focused on emissions from filament 3D printers.

**Results:**

VOC emissions from resin 3D printers have been reported mostly as carbonyl compounds or methacrylate monomers. Filament VOC emissions are more varied in composition reflecting the constituents in the filaments used in this printer. The published research reported that the airborne concentrations of specific VOCs from 3D desktop printers fell below the HSE British workplace exposure limits (WELs). This may suggest that VOC emissions from these printers do not present a risk to occupational health. However, caution is required in reaching this conclusion because most of these studies quantified specific VOC emissions using methods different to those required by workplace regulatory standards. Other exposure circumstances, such as the effect of total VOC emissions, need to be considered, particularly for vulnerable groups, including individuals with respiratory disease, the elderly, or young children. Variables that could increase exposure and risks to health include long print times, multiple 3D printers, and poor ventilation. Research on the VOC emissions from resin 3D printers is required using experimental emission chambers.

**Impact:**

- The research discussed in this review focused on VOC emissions from desktop 3D printers and the potential health impacts associated with exposure to these compounds. The review identifies circumstances when people may be exposed to 3D printer emissions for which no regulatory exposure limits apply. This circumstance is especially relevant to people working in small businesses and organisations and to vulnerable people, such as the young, elderly and those with pre-existing lung disease. Raising awareness of these potential health concerns from 3D printer emissions can help to inform actions to mitigate exposure, through policy and behavioural changes, as well as engineering control measures. To our knowledge, this is the first review discussing studies of VOC emission from resin and popular filament 3D printers, including exposure risks and health outcomes.

## Introduction

Over the past few decades, three-dimensional (3D) printing has advanced both technically and commercially. Desktop 3D printers are now used in schools, hospitals [1], dental practices [2], small offices, libraries [3] and inside homes [4], with potential risks to human health from printer emissions.

Multiple types of 3D printers use varied materials and different printing methodologies. Material extrusion (ME) is a common type of 3D printing, and fused deposition modelling (FDM)^TM^, and fused filament fabrication, are types of ME [5]. This uses a solid thermoplastic filament which is melted and extruded as droplets to build the object in layers [6, 7]. Another printing process called vat photopolymerization (VP) employs a reservoir of unpolymerized liquid resin which is selectively polymerised (cured) using light from a laser or LED source [5, 8–10]. VP includes stereolithography (SLA), digital light processing (DLP) and liquid crystal display (LCD).

Volatile organic compounds (VOCs) are chemicals in the air that are inhaled during breathing. Total VOC (TVOC) is the total amount of VOC that can be made up from various natural and man-made sources. The TVOC encompasses all the VOCs in the air.

Poor air quality can affect human health. Hazardous gases and small particles that penetrate the lungs and may cross the mucosal membrane to be absorbed into the bloodstream [11, 12]. Additionally, particles and aerosols, such as condensed droplets, may damage the lung’s lining causing inflammation and damage [13]. VOC emissions have been associated with an increased risk of developing pulmonary disease alongside other factors such as the age of the exposed person and the VOC they were exposed to [14]. Further research has linked exposure to certain VOCs to oxidative stress, decreased lung function, rhinitis, and early airway obstruction [15]. Exposure to VOCs has also been linked with an increased risk of a variety of cancers [16–18], due to many VOCs exhibiting carcinogenic effects [19], such as lung cancer [20] or leukaemia [21].

The VOC type and the exposure duration and dose affect human health. High exposures to VOCs can cause acute health effects such as respiratory irritation or wheezing. While repeated exposures to VOCs in high concentrations may cause chronic health impacts, including respiratory sensitisation or worsening or asthma or COPD.

Previous studies of 3D printers have identified emissions of several hazardous VOCs, such as styrene [7, 22–31] methyl methacrylate [22, 29, 32, 33] isopropanol [32], benzene [22–24, 34], and toluene [22–24, 28, 30, 34]. Some of these VOCs have been linked to adverse health consequences after large exposure doses, including increased risk of some cancers [16–21]. Health risks may arise from the printing process, the print materials, the final printed item, and any post-printing processes employed [3, 19, 28, 30, 35–38]. Measurements for these studies were varied, with minimal overlap in methodology. For example, Farcas et al. used evacuated gas canisters which were run through GCMS [23], while Mendes et al. used a variety of gaseous collection methods including Tenax TA 5TD tubes coupled to TD-GC/MS and a photo ioniser detector for volatiles [7]; This review focuses on evidence regarding VOC emissions from resin bed VP 3D printers and considers emerging evidence about VOC emissions from ME 3D printers. VP printers use lasers to cure monomers (e.g., methacrylate or acrylate molecules) using photo-initiators (e.g., diphenyl (2,4,6-trimethylbenzoyl) phosphine oxide [39]) which absorb the energy and start a polymerisation reaction [10] to form the printed object. These beds of liquid monomers, photo-initiators and other additives are covered with an ultra-violet light (UV) filter hood, which is not airtight and only protects the resin for curing in ambient light. Since the resin beds are not sealed, they can release VOCs into the environment. ME 3D printers heat thermoplastics such as PLA (polylactic acid) or ABS (acetonitrile-butadiene-styrene) to a melting point and then extrude this in layers to form the desired object shape. Printing with plastic filaments has also been found to release VOCs, including potentially harmful chemicals such as formaldehyde, acetaldehyde, styrene, benzene and toluene [7, 22–24, 26, 33, 34, 40, 41].

This review builds on previous research into VOC and particle emissions from ME printers which have been found to release various emissions [7, 22–24, 26, 33, 34, 40, 41] including reactive chemicals associated with health risks.

This review focuses on desktop 3D printers, not industrial 3D printers. The use of desktop printers by hobbyists, schools, and small businesses has grown recently and end users need support to use them safely.

This article covers stereolithographic 3D printing VOC emissions and their quantification. Studies of VOC emissions from ME printing with ABS and PLA, two of the more common filaments, are also included in the review because most of the research on VOC and particle emissions has been carried out for these materials. The potential health implications of the identified VOCs are also discussed.

## Methodology

A table of search terms was created for the 3D printer emissions literature searches. These terms, and synonyms, were used to search the Web of Science and PubMed databases and Google Scholar which identified 1861 ‘candidate’ papers. The search was carried out in April 2024. These candidates were filtered using the inclusion/exclusion criteria designed to identify papers which focused on VOC emissions from VP, or ME 3D printing using either ABS or PLA, filaments, where the VOC emissions were included. Binder jet resin 3D printers were not considered during this study as the focus was on vat photopolymerisation methods of resin 3D printing. The inclusion criteria are shown in Table 1. The remaining studies on VOC emissions from resin-bed and filament desktop 3D printers were also assessed. Forty-seven papers were included in this review as they were relevant to the study of VOC emissions and desktop 3D printing, Table 2, while only thirteen papers included VOC quantification emissions. Papers that only assessed particulate emissions or 3D printing pens were excluded from this review. The methodologies within the previous literature papers identified in this review were different across studies, leading to non-standardised measurements and difficulties in comparing data. For example, air changes and the room or chamber sizes varied for most papers, and different VOC samplers were used TVOC, real time measurements and air samples were deployed to identify VOC concentrations or emission rates. This lack of standardisation is a limitation of the current research field as reported VOC concentrations may also vary due to different methodologies.

**Table 1:** The inclusion and exclusion criteria for the literature sift stage after the literature search.

Inclusion Criteria	Exclusion criteria
Studies which are published in international scientifically peer-reviewed journals, or by international expert bodies (government or standards bodies)	Studies published before 1990
Studies on 3D printers based on the use of liquid resin bed or polymer filaments	Studies of other printers (i.e., not 3D printers)
Studies which are based on the use of VP and or ME 3D printers	Studies where the abstract is also not in English
Studies which focus on emissions from the 3D printers (VOC, particles and other volatile gases and ions)	Studies which contain no methodology or use of replicate samples
Studies which quantified emissions based on sample replicates and described the methodology	Studies which only evaluated particulate emissions
Studies included Volatile Organic Compound emissions from 3D printers	Material extrusion studies where ABS or PLA are not mentioned

**Table 2:** The stages of the paper sift during the review of previous literature and the total number of included papers in this review.

	Papers rejected	Papers remaining
All papers		1861
Eliminate papers before 1990	0	1861
Not in English	0	1861
Eliminate papers by inclusion/exclusion criteria	1814	47
Papers selected		47
Quantified VOC papers selected		13

### Novel insights and importance

The novelty of this paper is the focus on VOC quantification studies for vat photopolymerization and material extrusion printers. To the authors’ knowledge, this is the first review which includes the quantified VOCs from resin-based 3D printers and examines resin-based 3D printers in significant detail. Most previous literature focused on ME; others have undertaken a general assessment of emissions from different types of 3D printers; or considered particulate emissions rather than VOC emissions. This review aims to bring together evidence from studies of VOC emissions from resin 3D printers, and two popular filaments from ME 3D printers, to assess the potential risks to the health of the operators.

### Interpretation of VOC emission data

The experimental emissions data in the reviewed papers have been summarised alongside the UKHSA (UK Health Security Agency) indoor air quality guideline values and the Health and Safety Executive (HSE) Workplace Exposure Limit (WEL) values for occupational exposure, which are different [42]. There are eleven published UKHSA values for specific hazardous VOCs measured in the air over a defined period; these are not measures of personal exposure [43]. The HSE WEL values are for VOCs in the air, averaged over a specified period, referred to as a time-weighted average (TWA). The WEL airborne concentrations require either sampling the substance in the worker’s breathing zone (i.e., personal sampling) or the workplace air (i.e., area monitoring). In Great Britain (GB), WELs are set by the HSE and outlined in the Control of Substances Hazardous to Health (COSHH) [42] and include limit values for specific VOCs within the workplace (Table 3).

**Table 3:** VOC exposure guideline values from Health and Safety Executive (HSE) 8-h workplace exposure limits, occupational safety and health administration (OSHA), and National Institute for Occupational Safety and Health (NIOSH).

	HSE, ppmv	HSE, mgm^−3^	OSHA. ppm	NIOSH, ppm
2-Hydroxypropyl acrylate	0.5	2.7		
Benzene	1	3.25	1	0.1
Formaldehyde	2	2.5	0.75	0.016
Methacrylic acid	20	72		
Methyl methacrylate	50	208		
Phenol	2	7.8	5	5
Propan-1-ol	200	500		
Propan-2-ol^a^	400	999		
Styrene	100	430	50	50
Toluene	50	191	10	100
Xylenes	50	220	100	100

Guidelines given for the VOCs identified from this review and similar compounds as a reference for exposure limits [42, 91] ppmv – parts per million by volume, mgm^−3^ – milligrams per cubic metre.

^a^Propan-2-ol is also referenced as isopropanol in the text.

The HSE WEL limit values are concentrations which must not be exceeded and can represent adequate control of the compounds within the air. This does not mean that concentrations below the WEL are safe as additional factors may increase the potential adverse outcomes, such as pre-existing health conditions or disabilities. For carcinogens (e.g., formaldehyde) and chemicals that cause asthma, duty holders must reduce exposure to ‘as low as reasonably practicable” (ALARP) since safe levels for these hazardous chemicals cannot be defined. The ALARP concentration is lower than the WEL value and should reflect industry standards for good control practice, which should continue to improve with time. The UKHSA guidelines for the eleven hazardous VOCs are only guidelines and are not enforced [43].

### Standards for monitoring exposure to VOCs

Exposure to VOCs is commonplace but can be raised in certain environments particularly indoors where reduced ventilation can lead to localised raised concentrations [44]. To regulate human exposure, regulatory and advisory bodies have published guidance values and limits for VOCs to protect the public and employees. The UKHSA eleven VOCs of concern were identified from a review of both literature and guidance published from 2000–2018 by other governments and countries. The guidelines for VOC concentrations differ, with some being 30 min, and other accumulated one-year averages. These differences allow VOC exposures to be averaged over long periods and give allowances for higher exposures, though only for short time periods. These differences in measurement time make it challenging to compare concentrations of different VOCs.

The WELs, Table 3, are especially important when the workplaces contain sources of VOCs, such as, machinery, engines, and chemical stocks. WELs are averaged over 15 min for short-term exposure or over an 8-h workday shift. They define good control practices to minimise risks to workers’ health. Short-term 15-min exposure limits are relevant to acute exposures which can cause mucosal irritation. WELs do not apply to the personal use of 3D printers in the home but have been included in this review as examples of exposure values set to minimise risks to health.

## Summary and discussion of evidence

### Vat photopolymerisation 3D printers

The printed object is created from a vat of liquid photopolymer by selectively curing using a UV laser source [45, 46] in either top-down (free surface SLA) [10, 47] or bottom-up (constrained surface SLA) [10] orientations. During the curing process, the liquid photopolymer is converted into a rubber-like material, and [48]. The printed structure is then washed in alcohol to remove uncured resin on the surface of the build and fully cured under UV light to solidify the resin. Depending on the type and model of the resin printer, the vat of liquid resin may be left in place, depending on the type and model of resin printer. All these steps may impact VOC emissions from the printer and lead to exposure within the immediate environment.

The design of resin beds may promote the evolution of VOCs into the environment. The printers use a liquid bed comprised of multiple compounds, including polymer monomers, photo-initiators which catalyse the process, and other additives mixed into the stock solution. This bed is designed to be a liquid at room temperature rather than ME printing, where a solid filament is melted. The resin in the liquid state therefore requires less energy for the constituent molecules to be emitted as gas vapour than the solid phase thermoplastic. When a cover is placed around a resin printer this may also influence the emission of VOCs into the environment. The cover is usually included as a UV shield over the resin bed, preventing the resin bed from curing in ambient light. This is not an airtight seal but may reduce ventilation around the resin bed, and lower VOC emissions into the immediate environment [49] until it is removed by the operator who may then be subject to transient higher exposures of VOCs [49].

The laser light does not cause widespread heating within the liquid resin bed during the light activated processes. Typically, in VP printers the resin bed is heated to around 30–40 °C which is considerably lower than the temperatures required to melt the thermoplastics in ME printing, in addition to the heated ME build plate [5].

In VP printing, the printed structures are washed with isopropanol and undergo a further curing stage post-printing, a step not often required for ME printing. VOC emissions from VP printers may be enhanced by the mechanical movement of the machine itself and other emissions may include fine metallic particles [50, 51].

### Composition of resins used for vat polymerisation

#### Types of resin

The type of resin used for the build determines the properties of the final structure. Therefore, the composition of the resins can be altered with additives to achieve the desired properties, for example, increased heat resistance, durability, or flexibility [10]. Some flexible polymers can be made from elastomeric polyurethane or flexible polyurethane [10].

However, the resin used for VP needs to remain relatively non-viscous as the resin layer needs to be renewed during the build [47, 52]. The higher viscosity of the resin has been linked with increased printing time as the liquid may be slower to reform the required layer of resin within the vat between curing each building layer [52].

#### Additives in the resins

Within the resin, there are additional compounds which aid the polymerisation process. There are the general monomers and oligomers [53, 54] which are polymerised into the structure and can account for up to 40% of the resin liquid [45], (e.g., methacrylate or acrylate molecules). In addition, up to 10% of the liquid base are photo-initiators (e.g., diphenyl(2,4,6-trimethylbenzoyl) phosphine oxide) which absorb UV, or visible, light releasing either free radicals or cations forming new reactive species leading to cross polymerisation [47, 53, 55]. Lastly, binding agents are added to help the resin layers form a cohesive structure, and these can account for 50–80% of the liquid resin base [45]. The curing process of resins is irreversible [10]. The addition and concentration of different additives change the chemical composition of the resin, and therefore the composition of the VOC emissions from the resin vat.

### Evidence of emissions from desktops 3D printers

#### VOC emissions from VP printers

The emissions from resin bed printers have been less widely researched. Table 4 summarises the results of some of the first studies published in this area and the type of compounds identified and quantified during printing.

**Table 4:** Volatile organic compounds quantified for resin bed 3D printers from the four identified studies during the literature search [32, 38, 56, 92] Concentrations are given as µg/m^3^ - micrograms per cubic metre or as µg/h – micrograms per hour.

VOC	Study	Emission rate, clear resin, unspecified manufacturer, µg/h, approximate	Concentration (range), clear resin, Formlabs µg/m^3^	Concentration (range), castable wax resin, Formlabs µg/m^3^	Concentration (Standard deviation), Formlabs grey photopolymer resin µg/m^3^	Measurement conditions	Health impacts
2-Butanone	Väisänen, 2022		6 µg/m^3^	<1 µg/m^3^		52 m^3^ 3D printing lab	Nose, throat and eye irritations
2-Ethylpiperazine	Väisänen, 2022			4 (4–5) µg/m^3^		52 m^3^ 3D printing lab	Non-hazardous
2-Hydroxyethyl methacrylate	Zhang [36]	Print ~330 µg/h				1 m^3^ chamber	Skin, and respiratory irritants
2-Hydroxypropyl methacrylate	Väisänen, 2022		6 (2–8) µg/m^3^			52 m^3^ 3D printing lab	Skin and respiratory irritant
2-Hydroxypropyl methacrylate	Bowers, [38]				SLA - 18.1–21.9 µg/m^3^ DLP - 31.2 (1.5) µg/m^3^	12.85 m^3^ steel chamber	Skin and respiratory irritant
2-Hydroxypropyl methacrylate	Zhang [36]	Print −2800 µg/h Wash - 1800 µg/h Cure - 1200 µg/h				1 m^3^ camber	Skin and respiratory irritant
2-Methyl-2,4-pentanediol	Zhang [36]	Wash ~40 µg/h				1 m^3^ chamber	Skin and eye irritation, reproductive toxicity
2-Pentanol, 4-methyl	Zhang [36]	Wash ~50 µg/h				1 m^3^ chamber	Severe eye damage, respiratory organ toxicity
2,6-Di-tert-butyl-4-methylphenol	Zhang [36]	Wash 60 µg/h Cure ~100 µg/h				1 m^3^ chamber	Toxic to aquatic life
Acetaldehyde	Väisänen, 2022		2 µg/m^3^	1 µg/m^3^		52 m^3^ 3D printing lab	Eye irritation, carcinogen, germ cell mutagen, respiratory organ toxicity
Acetaldehyde	Bowers, [38]				SLA - 10.0–16.3 µg/m^3^ DLP - 8.3 (1.4) µg/m^3^	12.85 m^3^ steel chamber	Eye irritation, carcinogen, germ cell mutagen, respiratory organ toxicity
Acetone	Väisänen, 2022		3 µg/m^3^	2 µg/m^3^		52 m^3^ 3D printing lab	Eye irritation, central nervous system toxicity, drowsiness
Acetone	Zisook [56]		245 ppbv =581 µg/m^3^			28 m^3^ sampling room	Eye irritation, central nervous system toxicity, drowsiness
Acetone	Bowers, [38]				SLA - 2.7–10.1 µg/m DLP - 13.6 (1.5) µg/m^3^	12.85 m^3^ steel chamber	Eye irritation, central nervous system toxicity, drowsiness
Acetone	Zhang [36]	Print ~280 µg/h				1 m^3^ chamber	Eye irritation, central nervous system toxicity, drowsiness
Alpha pinene	Väisänen, 2022			6 (5–7) µg/m^3^		52 m^3^ 3D printing lab	Oral toxicity, skin irritation, aquatic hazard
Benzaldehyde	Väisänen, 2022		<1 µg/m^3^	<1 µg/m^3^		52 m^3^ 3D printing lab	Acute toxicity, skin and eye irritation, reproductive toxicity, respiratory toxicity
Butyraldehyde	Väisänen, 2022		4 µg/m^3^	3 µg/m^3^		52 m^3^ 3D printing lab	Eye irritation, flammable
Crotonic anhydride	Zhang [36]	Print ~180 µg/h				1 m^3^ chamber	Skin corrosive
Dodecane,2,6,11-trimethyl	Zhang [36]	Cure ~350 µg/h				1 m^3^ chamber	Aquatic hazard
Ethanol	Bowers, [38]				SLA - 0.02−4.5 µg/m^3^ DLP - 7.4 (3.2) µg/m^3^	12.85 m^3^ steel chamber	Eye irritation, flammable
Ethyl methacrylate	Väisänen, 2022		4 (4–5) µg/m^3^			52 m^3^ 3D printing lab	Skin corrosion, eye damage, respiratory toxicity
Formaldehyde	Väisänen, 2022		1 µg/m^3^	1 µg/m^3^		52 m^3^ 3D printing lab	Fatal if swallowed, skin corrosion, carcinogen, germ cell mutagen, organ toxicity
Hexaldehyde	Väisänen, 2022		4 µg/m^3^	1 µg/m^3^		52 m^3^ 3D printing lab	Skin and eye irritation
Isopropanol	Zisook [56]		560 ppbv =1374 µg/m^3^			28 m^3^ sampling room	Eye irritation, respiratory toxicity
Isopropanol	Zhang [36]	Wash 87729 µg/h Cure ~1100 µg/h				1 m^3^ chamber	Eye irritation, respiratory toxicity
Methylene chloride	Bowers, [38]				SLA - 0.03–16.0 µg/m^3^ DLP - 3.4 (5.4) µg/m^3^	12.85 m^3^ steel chamber	Skin and eye irritation, carcinogen, central nervous system toxicity
Methyl-methacrylate	Väisänen, 2022		6 (5–7) µg/m^3^			52 m^3^ 3D printing lab	Skin irritation, respiratory toxicity
Nonanal	Väisänen, 2022			13 (11–14) µg/m^3^		52 m^3^ 3D printing lab	Skin irritation
Propionaldehyde	Väisänen, 2022		1 µg/m^3^	1 µg/m^3^		52 m^3^ 3D printing lab	Oral toxicity, skin corrosion, respiratory toxicity
Propylene glycol	Zhang [36]	Print ~350 µg/h				1 m^3^ chamber	Non-hazardous
Styrene	Bowers, [38]				SLA - <1.52 µg/m^3^ DLP - 4.5 µg/m^3^	12.85 m^3^ steel chamber	Acute toxicity, reproductive toxicity, respiratory and hearing organ toxicity, fatal if swallowed
Undecane, 3,7-dimethyl	Zhang [36]	Cure ~60 µg/h				1 m^3^ chamber	Unknown
Total carbonyl	Väisänen, 2022		21 µg/m^3^			52 m^3^ 3D printing lab	Additive effects from all VOCs present
TVOC	Väisänen, 2022		41 (32–48) µg/m^3^	46 (43–51) µg/m^3^		52 m^3^ 3D printing lab	Additive effects from all VOCs present

Health impacts have been taken from the safety data sheets for each compound.

Four papers were identified from the literature search which quantified VOC emissions from resin-based 3D printers. Additional papers were identified that reported TVOC emission peaks and factors impacting the emissions, though these did not include tabulated data.

*SLA* stereolithography 3D printer used, *DLP* digital light processing.

Stereolithography describes the process in which an object is made by adding layer upon layer by using a laser to selectively cure resin into shape on the bottom of the structure, before the subsequent layer is formed. The laser scans the entire plane of the structure, focusing the light on the selected curing zone for each pass. Previous research by Väisänen et al. [32] identified and quantified a range of carbonyl compounds released during stereolithographic printing a Formlabs Form 2, resin 3D printer with clear and castable Formlabs wax resins. Dental resins were also examined. The samples were collected onto Tenax TA sorbent tubes collecting 6 l of air, from 1 m away at 1.5 m high. The samples were analysed using Thermo-Desorption Gas Chromatography Mass Spectrometry (TD-GC-MS).

The three most abundant VOCs identified by Väisänen [32] for each resin were identified and quantified, and an additional set of VOCs were quantified for each resin. The most abundant VOCs for the clear resin were 2-hydroxypropyl methacrylate (6 µg/m^3^), ethyl methacrylate (4 µg/m^3^) and methyl methacrylate (6 µg/m^3^); whilst for the castable wax, the highest emission was found for 2-ethyl piperazine (4 µg/m^3^), alpha-pinene (6 µg/m^3^) and nonanal (13 µg/m^3^). The three methacrylate compounds identified from the clear resin were listed as an unspecified methacrylate monomers mixture in the Formlabs SDS sheet [39] to maintain commercial confidentiality. [39] For the clear and wax resins the majority of the VOCs were emitted at the same or similar concentrations, around 1–5 µg/m^3^. The larger difference was for 2-butanone, where the clear and wax emitted 6 and <1 µg/m^3^, respectively.

The most abundant VOCs identified by Väisänen, from the four dental resins tested, emitted a set of similar compounds: isopropyl alcohol (15–22 µg/m^3^), methyl isobutyl ketone (7–24 µg/m^3^), tert-butyl alcohol (12–17 µg/m^3^), with the addition of 2-ethoxypropane (8 µg/m^3^) and 1,3-dioxolane (7 µg/m^3^). All the dental resins emitted high concentrations of acetone, from 25–37 µg/m^3^. These were the highest reported emissions from these four dental resins.

In previous research led by Zisook [56], four types of 3D printers were tested, including an SLA VP printer. A 3D systems 3D printer from ProJet 3000 was used for VP and sited in a 28 m^2^ room. VOC samples were collected into a MiniCan VOC sampler located 0.3–0.7 m from the printer. The samples were analysed using GC-MS, and real time VOC monitoring was also undertaken.

The resin printer released acetone and isopropanol VOCs at higher concentrations (245 and 560 ppbV) than the background (control) samples (2 and 47% higher than their background respectively) [56] The isopropanol emissions were 560 ppbV (0.560 ppmV) compared to the occupational TWA exposure limit of 400 ppmV [57]. The acetone emissions from the printing were 245 ppbV (0.245 ppmV) compared to the WEL of 500 ppmV [57]. The reported concentrations from the printers measured over a few minutes and therefore may not be representative of the 8-h averages against which they are compared (GB WELs). The difference between concentrations and WELs showed that the printer emissions were around three orders of magnitude smaller than the exposure limit. Propylene and toluene were both below detection limits (5 ppbV for both detection limits).

Acetone was quantified by all four studies, ranging from 2 µg/m^3^ [32] to 245 ppbV (580 µg/m^3^) [56]. There is almost a 200-fold difference between these two measurements. This may be due in part to the conditions of the experimental work. The exposure rooms were different sizes, and the sampling equipment was placed in several places (1.5 m high, 1 m away from the printer; and breathing height, adjacent to the printer).

Previous research carried out by Krechmer et al. [58], measured the differences in outgassing of VP printed components for both clear and dental resin, in both cured and uncured states. The study identified several chemical structures, methyl acrylate, methyl methacrylate, ethyl methacrylate, and propyl methacrylate; along with benzene, xylenes, alkylated benzenes, cyclopentene/isoprene, other terpenes, formaldehyde, and formic acid. As time-of-flight mass spectrometry (TOF-MS) is not able to identify isomers, the chemical formula was used to identify the VOCs. Acrylonitrile was also identified but attributed to the gloves worn whilst touching the printed structures. The curing stage of post-processing is critical to reducing VOC emissions up to ten-fold [58]. The printed items were also found to outgas the identified chemicals for several hours, and the data was fitted to an exponential decay pattern. The authors suggested leaving any printed items in a gas flow for 2–3 h to reduce VOC emissions considerably [58].

In previous research led by Yang [49], an emission model based on VP printing was proposed and experimentally validated. The study considered the contribution of the manufacturing process, and the liquid volatilisation process of the VOCs into the laboratory air, in addition to any post-processing involved. TVOC emitted during volatilisation only was calculated to be 106.504 µg/m^3^, and the recorded value was 122.70 µg/m^3^, resulting in a 14% error. During printing, the mean TVOC concentration was recorded at 1052.71 µg/m^3^, and the post-processing cure and ethanol wash emitted a mean TVOC of 1774.15 µg/m^3^ [49]. The authors also investigated two emission mitigation methods: titanium dioxide photocatalytic oxidation and activated carbon absorption. Both were found to reduce TVOC during active printing by 44–71% [49], respectively.

Previous research led by Vasilescu investigated the printing and post-processing of a resin-bed 3D printer for both formaldehyde and TVOC [59]. Both formaldehyde and TVOC increased during the printing process non-linearly and peaked during post-processing. The maximum TVOC recorded was 9.999 mg/m^3^, 30-fold higher than during printing (0.363 mg/m^3^). These measurements used a JBLB600 multifunctional air quality monitor, and the peak values may represent the upper detection limit, not necessarily the highest concentration present. They also recommended increasing ventilation and using air filtration during and after the printing process, which further supports Krechmer et al. where the additional time under a gas flow was also recommended [58].

#### VOC emissions from ME printers

Published studies have shown that the type of ME printer and filament material used directly impacts the type and quantity of VOC emissions. Many of the published studies also indicate that the types of ME feedstock filament materials affect VOC emissions. ME 3D printers and the filaments ABS and PLA; have been the subject of most research into emissions and air quality. Table 5 and Table 6 summarise the identification and quantification of VOCs emitted from these ME printers, separated by filament used and concentration and emission rate.

**Table 5:** Volatile organic compounds quantified from studies of material extrusion 3D printers using ABS polymer filaments [7, 22, 23, 26, 34] and PLA filaments [7, 22, 24, 34, 93].

Compound	Filament	Study	Concentration	µg/m^3^ standardised units	Type of measurement	Health impacts of the VOC
1,3-Butadiene	ABS	Kim et al. 2022	8.4 ppb	18.58 µg/m^3^	206.55 m^3^ sampling room	Germ cell mutagen
2,3-Hexenedione	ABS	Farcas et al. [23]	0.0011 ppm	5.14 µg/m^3^	whole body, rodent exposure chamber	Skin corrosive, respiratory toxicity
2-propenol	ABS	Gu, J. W. et al. [26]	4 µg/m^3^	4 µg/m^3^	3 m^3^ chamber exposure	Eye irritation, nervous system toxicity
Acetaldehyde	ABS	Kim et al. [34]	57.5 µg/m^3^	57.5 µg/m^3^	1 m^3^ test chamber	Eye irritation, carcinogen, germ cell mutagen, respiratory organ toxicity
	ABS	Gu, J. W. et al. [26]	12 µg/m^3^	12 ug/m^3^	3 m^3^ chamber exposure	
	ABS	Yi et al. [22]	<background - 393.6 µg/m^3^	<background - 393.6 µg/m^3^	0.6 m^3^ chamber exposure	
	ABS	Kim et al. 2022	8.4 ppb	15.13 µg/m^3^	206.55 m^3^ sampling room	
	ABS	Farcas et al. [23]	0.1105 ppm	200 µg/m^3^	whole body, rodent exposure chamber	
Acetone	ABS	Gu, J. W. et al. [26]	5.5 µg/m^3^	5.5 µg/m^3^	3 m^3^ chamber exposure	Eye irritation, central nervous system toxicity,
	ABS	Yi et al. [22]	3.1–18.2 µg/m^3^	3.1–18.2 µg/m^3^	0.6 m^3^ chamber exposure	
	ABS	Farcas et al. [23]	0.0112 ppm	26.61 µg/m^3^	whole body, rodent exposure chamber	
	ABS	Kim et al. 2022	1493.6 ppb	3,548 µg/m^3^	206.55 m^3^ sampling room	
Acrolein	ABS	Kim et al. 2022	6.3 ppb	14.44 µg/m^3^	206.55 m^3^ sampling room	Oral, inhalation and dermal toxicity, skin corrosion, eye damage
Acrylonitrile	ABS	Gu, J. W. et al. [26]	2 µg/m^3^	2 µg/m^3^	3 m^3^ chamber exposure	Oral, inhalation and dermal toxicity, carcinogen, respiratory toxicity
	ABS	Farcas et al. [23]	0.0045 ppm	9.77 µg/m^3^	whole body, rodent exposure chamber	
Benzene	ABS	Kim et al. [34]	<LOD	<LOD	1 m^3^ test chamber	Skin and eye irritation, germ cell mutagen, carcinogen, blood toxicity
	ABS	Yi et al. [22]	1.1–1.5 µg/m^3^	1.1–1.5 µg/m^3^	0.6 m^3^ chamber exposure	
	ABS	Farcas et al. [23]	0.0036 ppm	11.5 µg/m^3^	whole body, rodent exposure chamber	
	ABS	Kim et al. 2022	0.4 ppb	1.28 µg/m^3^	206.55 m^3^ sampling room	
Chloroethylene	ABS	Kim et al. 2022	1.1 ppb	2.79 µg/m^3^	206.55 m^3^ sampling room	Carcinogen
Chloroform	ABS	Kim et al. 2022	3.3 ppb	16.1 µg/m^3^	206.55 m^3^ sampling room	Oral and inhalation toxicity, skin corrosive, carcinogen, reproductive toxicity, nervous system, liver and kidney toxicity
D-limonene	ABS	Farcas et al. [23]	0.003 ppm	16.72 µg/m^3^	whole body, rodent exposure chamber	Skin corrosion, eye damage, reproductive and aspiration toxicity
Dimethyl disulfide	ABS	Kim et al. 2022	2.4 ppb	9.24 µg/m^3^	206.55 m^3^ sampling room	Toxic if inhaled or swallowed, respiratory and nervous system toxicity, skin sensitization
Ethanol	ABS	Gu, J. W. et al. [26]	2.5 µg/m^3^	2.5 µg/m^3^	3 m^3^ chamber exposure	Eye irritation, flammable
	ABS	Yi et al. [22]	<background - 187.5 µg/m^3^	<background - 187.5 µg/m^3^	0.6 m^3^ chamber exposure	
	ABS	Farcas et al. [23]	0.019 ppm	35.8 µg/m^3^	whole body, rodent exposure chamber	
	ABS	Kim et al. 2022	181.5 ppb	341.26 µg/m^3^	206.55 m^3^ sampling room	
Ethylbenzene	ABS	Kim et al. [34]	11.5 ppb	49.9 µg/m^3^	1 m^3^ test chamber	Acute toxicity, hearing organ toxicity, aspiration hazard
		Yi et al. [22]	368.7–647.3 µg/m^3^	368.7–647.3 µg/m^3^	0.6 m^3^ chamber exposure	
	ABS	Kim et al. 2022	1.3 ppb	5.63 µg/m^3^	206.55 m^3^ sampling room	
Formaldehyde	ABS	Kim et al. [34]	68.0 ppb	83.5 µg/m^3^	1 m^3^ test chamber	Fatal if swallowed, skin corrosion, carcinogen, germ cell mutagen, organ toxicity
		Kim et al. [34]	29.8 ppb	36.54 µg/m^3^	1 m^3^ test chamber	
Hexane	ABS	Kim et al. 2022	10.6 ppb	37.33 µg/m^3^	206.55 m^3^ sampling room	Skin irritation, reproductive toxicity, nervous system toxicity, aspiration hazard
Isopropanol	ABS	Yi et al. [22]	<background −19.3 µg/m^3^	<background −19.3 µg/m^3^	0.6 m^3^ chamber exposure	Eye irritation, respiratory toxicity
	ABS	Farcas et al. [23]	0.0034 ppm	8.36 µg/m^3^	whole body, rodent exposure chamber	
	ABS	Kim et al. 2022	66.3 ppb	162.87 µg/m^3^	206.55 m^3^ sampling room	
Isovaleraldehyde	ABS	Kim et al. [34]	90.8 ppb	319.8 µg/m^3^	1 m^3^ test chamber	Eye damage, skin sensitization, respiratory toxicity
Methylethylketone	ABS	Kim et al. 2022	4.3 ppb	12.67 µg/m^3^	206.55 m^3^ sampling room	Serious eye damage, nervous system, liver and kidney toxicity
Methanol	ABS	Kim et al. 2022	102.5 ppb	134.24 µg/m^3^	206.55 m^3^ sampling room	Acute oral, inhalation and dermal toxicity, eyes and nervous system organ toxicity
Methyl-methacrylate	ABS	Farcas et al. [23]	0.0037 ppm	15.15 µg/m^3^	whole body, rodent exposure chamber	Skin irritation, respiratory toxicity
PGME	ABS	Kim et al. 2022	9.4 ppb	34.62 µg/m^3^	206.55 m^3^ sampling room	Respiratory and nervous system organ toxicity
Phenol	ABS	Kim et al. 2022	8.2 ppb	31.54 µg/m^3^	206.55 m^3^ sampling room	Acute oral, dermal and inhalation toxicity, skin corrosion, germ cell mutagen, organ toxicity, toxic if swallowed
Propene	ABS	Kim et al. 2022	124.9 ppb	214.83 µg/m^3^	206.55 m^3^ sampling room	Flammable
Styrene	ABS	Yi et al. [22]	1318–2216 µg/m^3^	1318–2216 µg/m^3^	0.6 m^3^ chamber exposure	Acute toxicity, reproductive toxicity, respiratory and hearing organ toxicity, fatal if swallowed
	ABS	Mendes, 2017	2 µg/m^3^	2 µg/m^3^	81 m^3^ ventilated room	
	ABS	Farcas et al. [23]	0.0024 ppm	10.22 µg/m^3^	whole body, rodent exposure chamber	
	ABS	Kim et al. 2022	2.7 ppb	11.48 µg/m^3^	206.55 m^3^ sampling room	
Toluene	ABS	Kim et al. [34]	3.7 ppb	13.9 µg/m^3^	1 m^3^ test chamber	Skin irritation, reproductive and nervous system toxicity, aspiration hazard
	ABS	Yi et al. [22]	2.4–4.4 µg/m^3^	2.4–4.4 µg/m^3^	0.6 m^3^ chamber exposure	
	ABS	Farcas et al. [23]	0.0084 ppm	31.66 µg/m^3^	whole body, rodent exposure chamber	
	ABS	Kim et al. 2022	13.0 ppb	58.45 µg/m^3^	206.55 m^3^ sampling room	
Trimethylamine	ABS	Kim et al. 2022	13.1 ppb	31.65 µg/m^3^	206.55 m^3^ sampling room	Acute toxicity, skin irritation, eye damage, respiratory toxicity
Xylenes-mixture	ABS	Kim et al. [34]	0.6 ppb after, <LOD during	2.6 (0.9) µg/m^3^ after, <LOD during	1 m^3^ test chamber	Acute toxicity, skin and eye irritation, respiratory, hearing organ, liver, kidney and central nervous system toxicity
	ABS	Farcas et al. [23]	0.0024 ppm	10.42 µg/m^3^	whole body, rodent exposure chamber	
	ABS	Kim et al. 2022	1.3 ppb	5.64 µg/m^3^	206.55 m^3^ sampling room	
TVOC	ABS	Mendes, 2017	230–270 µg/m^3^	230–270 µg/m^3^ (background)	0.18 m^3^ exposure chamber	Combination of health impacts from present VOCs
	ABS	Mendes, 2017	250–520 µg/m^3^	250–520 µg/m^3^	81 m^3^ ventilated room	
	ABS	Kim et al. [34]	avg 154.9 ppb, top 453.3 ppb		1 m^3^ test chamber	
1,2,4-trichlorobenzene	PLA	Youn et al. [24]	0.26 ppb	1.93 µg/m^3^	126 m^3^ sampling room	Acute oral toxicity, skin corrosion and irritation, eye damage and irritation
1,4-dichlorobenzene	PLA	Youn et al. [24]	0.30 ppb	1.8 µg/m^3^	126 m^3^ sampling room	Eye damage and irritation, carcinogen
2-Butanone	PLA	Vaisanen et al. 2022	20 µg/m^3^	20 µg/m^3^	104 m^3^ sampling room	Nose, throat and eye irritations
Acetaldehyde	PLA	Kim et al. [34]	30.4 ppb	54.8 µg/m^3^	1 m^3^ test chamber	Eye irritation, carcinogen, germ cell mutagen, respiratory organ toxicity
			18.4 ppb	33.1 µg/m^3^	1 m^3^ test chamber	
	PLA	Vaisanen et al. 2022	6 µg/m^3^	6 µg/m^3^	104 m^3^ sampling room	
Acetic acid	PLA	Vaisanen et al. 2022	3 µg/m^3^	3 µg/m^3^	104 m^3^ sampling room	Skin corrosion, eye damage
Acetone	PLA	Yi et al. [22]	<background −22.4 µg/m^3^	<background −22.4 µg/m^3^	0.6 m^3^ chamber exposure	Eye irritation, central nervous system toxicity, drowsiness
	PLA	Vaisanen et al. 2022	25 µg/m^3^	25 µg/m^3^	104 m^3^ sampling room	
Acrylonitrile	PLA	Youn et al. [24]	3.36 ppb	7.29 µg/m^3^	126 m^3^ sampling room	Oral, inhalation and dermal toxicity, carcinogen, respiratory toxicity
Benzene	PLA	Kim et al. [34]	<LOD	<LOD	1 m^3^ test chamber	Skin and eye irritation, germ cell mutagen, carcinogen, blood toxicity
	PLA	Youn et al. [24]	~0.5 ppb	1.6 µg/m^3^	126 m^3^ sampling room	
	PLA	Yi et al. [22]	<background −1.4 µg/m^3^	<background −1.4 µg/m^3^	0.6 m^3^ chamber exposure	
Chlorobenzene	PLA	Youn et al. [24]	0.16 ppb	0.74 µg/m^3^	126 m^3^ sampling room	Acute toxicity
Chloroform	PLA	Youn et al. [24]	0.32 ppb	1.56 µg/m^3^	126 m^3^ sampling room	Oral and inhalation toxicity, skin corrosive, carcinogen, reproductive toxicity, nervous system, liver and kidney toxicity
Ethanol	PLA	Yi et al. [22]	<background - 73.9 µg/m^3^	<background - 73.9 µg/m^3^	0.6 m^3^ chamber exposure	Eye irritation, flammable
Ethylbenzene	PLA	Kim et al. [34]	0.8 ppb	3.5 µg/m^3^	1 m^3^ test chamber	Acute toxicity, hearing organ toxicity, aspiration hazard
			1.2 ppb	5.2 µg/m^3^	1 m^3^ test chamber	
	PLA	Yi et al. [22]	<background - 0.9 µg/m^3^	<background - 0.9 µg/m^3^	0.6 m^3^ chamber exposure	
Formaldehyde	PLA	Kim et al. [34]	54.0 ppb	66.3 µg/m^3^	1 m^3^ test chamber	Fatal if swallowed, skin corrosion, carcinogen, germ cell mutagen, organ toxicity
			155.9 ppb	191.5 µg/m^3^	1 m^3^ test chamber	
	PLA	Vaisanen et al. 2022	6 µg/m^3^	6 µg/m^3^	104 m^3^ sampling room	
Hexachloro1,3-butadiene	PLA	Youn et al. [24]	0.27 ppb	2.88 µg/m^3^	126 m^3^ sampling room	Acute oral, dermal and inhalation toxicity, skin corrosive, carcinogen, organ toxicity
Hexanal	PLA	Vaisanen et al. 2022	2 µg/m^3^	2 µg/m^3^	104 m^3^ sampling room	Skin and eye irritation
Hexane	PLA	Youn et al., [24]	8.46 ppb	29.82 µg/m^3^	126 m^3^ sampling room	Skin irritation, reproductive toxicity, nervous system toxicity, aspiration hazard
Isopropanol	PLA	Yi et al. [22]	<background - 62.5 µg/m^3^	<background - 62.5 µg/m^3^	0.6 m^3^ chamber exposure	Eye irritation, respiratory toxicity
Isovaleraldehyde	PLA	Kim et al. [34]	<LOD / 27.2 ppb	<LOD / 95.8 µg/m^3^	1 m^3^ test chamber	Eye damage, skin sensitization, respiratory toxicity
Lactide	PLA	Vaisanen et al. 2022	22 µg/m^3^	22 µg/m^3^	104 m^3^ sampling room	Skin corrosion, eye damage, respiratory toxicity
Methylene chloride	PLA	Youn et al. [24]	1.64 ppb	5.7 µg/m^3^	126 m^3^ sampling room	Carcinogen eye irritation, carcinogen, central nervous system toxicity
Methyl-methacrylate	PLA	Yi et al. [22]	<background - 1.7 µg/m^3^	<background - 1.7 µg/m^3^	0.6 m^3^ chamber exposure	Skin irritation, respiratory toxicity
Nonanol	PLA	Vaisanen et al. 2022	3 µg/m^3^	3 µg/m^3^	104 m^3^ sampling room	Skin irritation
Propanol	PLA	Vaisanen et al. 2022	2 µg/m^3^	2 µg/m^3^	104 m^3^ sampling room	Eye irritation, nervous system toxicity
Styrene	PLA	Youn et al. [24]	0.33 ppb	1.41 µg/m^3^	126 m^3^ sampling room	Acute toxicity, reproductive toxicity, respiratory and hearing organ toxicity, fatal if swallowed
	PLA	Yi et al. [22]	<background - 3.2 µg/m^3^	<background - 3.2 µg/m^3^	0.6 m^3^ chamber exposure	
Tetrachloroethylene	PLA	Youn et al. [24]	0.02 ppb	0.14 µg/m^3^	126 m^3^ sampling room	Skin corrosion, eye damage, carcinogen, nervous system, kidney, liver and blood toxicity
Toluene	PLA	Kim et al. [34]	16.2 ppb	61.0 µg/m^3^	1 m^3^ test chamber	Skin irritation, reproductive and nervous system toxicity, aspiration hazard
			2.7 ppb	5.7 µg/m^3^	1 m^3^ test chamber	
	PLA	Youn et al. [24]	1.65 ppb	6.22 µg/m^3^	126 m^3^ sampling room	
	PLA	Yi et al. [22]	<background - 0.2 µg/m^3^	<background - 0.2 µg/m^3^	0.6 m^3^ chamber exposure	
Trichloroethylene	PLA	Youn et al. [24]	~0.09 ppb	0.48 µg/m^3^	126 m^3^ sampling room	Skin and eye irritation, germ cell mutagen, carcinogen, nervous system toxicity
Xylenes-mixture	PLA	Kim et al. [34]	0.8 ppb	3.5 µg/m^3^	1 m^3^ test chamber	Acute toxicity, skin and eye irritation, respiratory, hearing organ, liver, kidney and central nervous system toxicity
			1.3 ppb	5.6 µg/m^3^	1 m^3^ test chamber	
	PLA	Youn et al. [24]	1.28 ppb	5.56 µg/m^3^	126 m^3^ sampling room	
TVOC	PLA	Mendes, 2017	250–520 µg/m^3^	250–520 µg/m^3^	81 m^3^ ventilated room	Combination of health impacts from present VOCs
	PLA	Yi et al. [22]	31.8 µg/g yellow		0.6 m^3^ chamber exposure	
			10.8 µg/g orange		0.6 m^3^ chamber exposure	

All concentrations have been converted to a standard unit to enable comparison between studies, µg/m^3^. Health impacts have been taken from the safety data sheets for each compound.

**Table 6:** emission rates of Volatile organic compounds emitted from material extrusion 3D printers using ABS filaments [7, 25, 26, 94] and PLA filaments [25, 94]. Health impacts have been taken from the safety data sheets for each compound.

Compound	Filament	Study	Emission rate	µg/min standardised units	Measurement	Health impact of VOC
Acetaldehyde	ABS	Davies et al. 2019	53.6 µg/h	0.89 µg/min	1 m^3^ exposure chamber	Eye irritation, carcinogen, germ cell mutagen, respiratory organ toxicity
Acetone	ABS	Wojtyla et al. 2017	1.6 × 10^−4^ µmol/min	0.00929 µg/min	thermal degradation of material in tube	Eye irritation, central nervous system toxicity, drowsiness
Acetophenone	ABS	Gu, J. W. et al. [26]	0.2 µg/min	0.2 µg/min	3 m^3^ chamber exposure	Acute toxicity, harmful if swallowed
		Davies et al. 2019	63.1 µg/h	1.05 µg/min	1 m^3^ exposure chamber	
Acrylonitrile	ABS	Gu, J. W. et al. [26]	0.2 µg/min	0.2 µg/min	3 m^3^ chamber exposure	Oral, inhalation and dermal toxicity, carcinogen, respiratory toxicity
Benzaldehyde	ABS	Gu, J. W. et al. [26]	0.8 µg/min	0.8 µg/min	3 m^3^ chamber exposure	Acute toxify, skin and eye irritation, reproductive toxicity, respiratory toxicity
		Davies et al. 2019	71.5 µg/h	1.19 µg/min	1 m^3^ exposure chamber	
Benzoic acid	ABS	Gu, J. W. et al. [26]	0.2 µg/min	0.2 µg/min	3 m^3^ chamber exposure	Skin irritation, serious eye damage, lung toxicity
Butadiene	ABS	Wojtyla et al. 2017	1.4 × 10^−3^ µmol/min	0.0757 µg/min	thermal degradation of material in tube	Germ cell mutagen
Butanol	ABS	Davies et al. 2019	19.8 µg/h	0.33 µg/min	1 m^3^ exposure chamber	Acute toxicity, skin irritation, eye damage, nervous and respiratory system toxicity
Cumene	ABS	Davies et al. 2019	18.1 µg/h	0.3 µg/min	1 m^3^ exposure chamber	Carcinogen, respiratory system toxicity, aspiration hazard
Cyclohexanone	ABS	Wojtyla et al. 2017	1.1 × 10^−3^ µmol/min	0.107 µg/min	thermal degradation of material in tube	Acute toxicity if swallowed or inhaled, skin irritation, respiratory system toxicity
Cyclotrisiloxane, hexamethyl	ABS	Davies et al. 2019	19.7 µg/h	0.328 µg/min	1 m^3^ exposure chamber	Flammable
Decanal	ABS	Davies et al. 2019	6.5 µg/h	0.108 µg/min	1 m^3^ exposure chamber	Serious eye irritation
Ethylbenzene	ABS	Wojtyla et al. 2017	2.2 × 10^−3^ µmol/min	0.234 µg/min	thermal degradation of material in tube	Acute toxicity, hearing organ toxicity, aspiration hazard
	ABS	Gu, J. W. et al. [26]	4.8 µg/min	4.8 µg/min	3 m^3^ chamber exposure	
		Davies et al. 2019	69.3 µg/h	1.155 µg/min	1 m^3^ exposure chamber	
Formaldehyde	ABS	Mendes, 2017	2–3 μg m^−3^, 30–40 ng s^−1^		0.18 m^3^ exposure chamber and 81 m^3^ ventilated room	Fatal if swallowed, skin corrosion, carcinogen, germ cell mutagen, organ toxicity
		Davies et al. 2019	24.7 µg/h	0.411 µg/min	1 m^3^ exposure chamber	
Isobutanol	ABS	Wojtyla et al. 2017	7.5 × 10^−4^ µmol/min	0.0556 µg/min	thermal degradation of material in tube	Skin irritation, eye damage, nervous and respiratory system toxicity
Phenol	ABS	Gu, J. W. et al. [26]	0.1 µg/min	0.1 µg/min	3 m^3^ chamber exposure	Acute oral, dermal and inhalation toxicity, skin corrosion, germ cell mutagen, organ toxicity, toxic if swallowed
Phensuximide	ABS	Davies et al. 2019	7.9 µg/h	0.131 µg/min	1 m^3^ exposure chamber	Harmful if swallowed
Propylbenzene	ABS	Davies et al. 2019	10.2 µg/h	0.17 µg/min	1 m^3^ exposure chamber	Respiratory system toxicity, aspiration toxicity
Styrene	ABS	Mendes, 2017	14 μg m^−3^, 200 ng s^−1^		0.18 m^3^ exposure chamber	Acute toxicity, reproductive toxicity, respiratory and hearing organ toxicity, fatal if swallowed
	ABS	Wojtyla et al. 2017	2.8 × 10^−3^ µmol/min	0.292 µg/min	thermal degradation of material in tube	
	ABS	Gu, J. W. et al. [26]	6.4 µg/min	6.4 µg/min	3 m^3^ chamber exposure	
	ABS	Davies et al. 2019	276 µg/h	4.6 µg/min	1 m^3^ exposure chamber	
Toluene	ABS	Davies et al. 2019	5.9 µg/h	0.098 µg/min	1 m^3^ exposure chamber	Skin irritation, reproductive and nervous system toxicity, aspiration hazard
Trichloroethane	ABS	Gu, J. W. et al. [26]	0.1 µg/min	0.1 µg/min	3 m^3^ chamber exposure	Acute oral, dermal and inhalation toxicity, carcinogen
Vinyl cyclohexene	ABS	Davies et al. 2019	20.4 µg/h	0.34 µg/min	1 m^3^ exposure chamber	Skin corrosive, carcinogen, reproductive and aspiration toxicity
Xylenes-mixture	ABS	Gu, J. W. et al. [26]	0.2 µg/min	0.2 µg/min	3 m^3^ chamber exposure	Acute toxicity, skin and eye irritation, respiratory, hearing organ, liver, kidney and central nervous system toxicity
		Davies et al. 2019	6.8 µg/h	0.113 µg/min	1 m^3^ exposure chamber	
1-Hexanol, 2-ethyl	PLA	Davies et al. 2019	0.6 µg/h	0.01 µg/min	1 m^3^ exposure chamber	Acute toxicity, skin and eye irritation, respiratory system toxicity
2,4-bis(1,1-dimethylethyl)-phenol	PLA	Davies et al. 2019	8.3 µg/h	0.138 µg/min	1 m^3^ exposure chamber	Acute oral toxicity, skin irritation and corrosive, serious eye damage, skin sensitization
Acetaldehyde	PLA	Davies et al. 2019	18.8 µg/h	0.313 µg/min	1 m^3^ exposure chamber	Eye irritation, carcinogen, germ cell mutagen, respiratory organ toxicity
Acetone	PLA	Wojtyla et al. 2017	1.9 × 10^−4^ µmol/min	0.011 µg/min	thermal degradation of material in tube	Eye irritation, central nervous system toxicity, drowsiness
Benzaldehyde	PLA	Davies et al. 2019	4.1 µg/h	0.068 µg/min	1 m^3^ exposure chamber	Acute toxify, skin and eye irritation, reproductive toxicity, respiratory toxicity
Butanol	PLA	Davies et al. 2019	17.8 µg/h	0.297 µg/min	1 m^3^ exposure chamber	Acute toxicity, skin irritation, eye damage, nervous and respiratory system toxicity
Calprolactam	PLA	Davies et al. 2019	7.4 µg/h	0.123 µg/min	1 m^3^ exposure chamber	Acute toxicity, skin and eye irritant, respiratory system toxicity
Cyclohexanone	PLA	Wojtyla et al. 2017	9.8 × 10^−4^ µmol/min	0.096 µg/min	thermal degradation of material in tube	Acute toxicity if swallowed or inhaled, skin irritation, respiratory system toxicity
Cyclotrisiloxane, hexamethyl	PLA	Davies et al. 2019	0.7 µg/h	0.011 µg/min	1 m^3^ exposure chamber	Flammable
Decanal	PLA	Davies et al. 2019	4.1 µg/h	0.068 µg/min	1 m^3^ exposure chamber	Serious eye irritation
Ethylbenzene	PLA	Davies et al. 2019	0.3 µg/h	0.005 µg/min	1 m^3^ exposure chamber	Acute toxicity, hearing organ toxicity, aspiration hazard
Formaldehyde	PLA	Davies et al. 2019	7.0 µg/h	0.117 µg/min	1 m^3^ exposure chamber	Fatal if swallowed, skin corrosion, carcinogen, germ cell mutagen, organ toxicity
Isobutanol	PLA	Wojtyla et al. 2017	1.5 × 10^−3^ µmol/min	0.111 µg/min	thermal degradation of material in tube	
Lactide	PLA	Davies et al. 2019	111 µg/h	1.85 µg/min	1 m^3^ exposure chamber	Skin corrosion, eye damage, respiratory toxicity
Methyl-methacrylate	PLA	Wojtyla et al. 2017	2.5 × 10^−3^ µmol/min	0.25 µg/min	thermal degradation of material in tube	Skin irritation, respiratory toxicity
		Davies et al. 2019	32.7 µg/h	0.545 µg/min	1 m^3^ exposure chamber	
Nonanal	PLA	Davies et al. 2019	2.9 µg/h	0.048 µg/min	1 m^3^ exposure chamber	Skin irritation
Styrene	PLA	Davies et al. 2019	1.6 µg/h	0.027 µg/min	1 m^3^ exposure chamber	Acute toxicity, reproductive toxicity, respiratory and hearing organ toxicity, fatal if swallowed
Tetradecane	PLA	Davies et al. 2019	0.7 µg/h	0.012 µg/min	1 m^3^ exposure chamber	Aspiration toxicity

When using the ABS filament, styrene was identified as one of the main thermoplastic components (up to 2216 µg/m^3^), [22] whilst PLA filaments released emissions of isovaleraldehyde (95.8 µg/m^3^) or methyl methacrylate (1.7 µg/m^3^) [60]. PLA has also been shown to release an acrylic acid dimer, caused by lactic acid dehydrating and dimerising, as well as d-limonene [27]. In contrast, some studies have found little to no increase in VOC emission during the operation of ME 3D printers [56].

The VOCs emitted during ME printing identified and quantified are shown in Tables 5 and 6. Several of these are listed by UKHSA amongst eleven indoor VOCs of concern: acetaldehyde was quantified up to 393.6 µg/m^3^ (UKHSA guideline for one hour is 1420 µg/m^3^, the GB WEL is 37 mg/m^3^), benzene up to 11.5 µg/m^3^ (UKHSA guideline gives no safe exposure value, the UK WEL is 3.25 mg/m^3^), ethanol up to 341.26 µg/m^3^ (the GB WEL is 1920 mg/m^3^), formaldehyde up to 83.5 µg/m^3^ (UKHSA guideline for 30 min is 100 µg/m^3^, the GB WEL is 2.5 mg/m^3^), styrene up to 2216 µg/m^3^ (UKHSA guideline for one year is 850 µg/m^3^, the GB WEL is 430 mg/m^3^), toluene up to 58.45 µg/m^3^ (UKHSA guideline for eight hours is 15,000 µg/m^3^, the GB WEL is 191 mg/m^3^) [42, 43]. All of the identified compounds were within the air quality guidance values, or the workplace exposure limits [42] assuming a continual exposure for the 8-h time averaged reference limits, however, styrene exceeded the UKHSA guidelines. The guidelines from the UKHSA relate to a year of exposure to styrene, which is unlikely to be reached by using a desktop 3D printer in a personal capacity. As the likelihood of an 8-h continuous exposure is low, adverse effects from short-term exposures appear unlikely. In addition, the VOC samplers were placed in a fixed position close to the emission source from either an emission chamber or emission room which may not be representative of a home user scenario.

During PLA printing, the concentrations were different, (see Table 5). Acetaldehyde was quantified at 54.8 µg/m^3^ (UKHSA guideline for one hour is 1420 µg/m^3^, the GB WEL is 37 mg/m^3^), benzene up to 1.6 µg/m^3^ (UKHSA guideline gives no safe exposure value, the GB WEL is 3.25 mg/m^3^), ethanol up to 73.9 µg/m^3^ (the GB WEL is 1920 mg/m^3^), formaldehyde up to 191.5 µg/m^3^ (UKHSA guideline for 30 min is 100 µg/m^3^, the GB WEL is 2.5 mg/m^3^), and toluene up to 61 µg/m^3^ (UKHSA guideline for eight hours is 15,000 µg/m^3^, the GB WEL is 191 mg/m^3^) [42, 43]. All compounds were within the workplace exposure limits [42], however, formaldehyde exceeded the UKHSA guideline. Two PLA filaments were tested, but only one exceeded the guidelines. The other PLA filament was within the UKHSA guidelines (66.3 µg/m^3^), showing variability between PLA filaments.

In addition to printing the filaments, VOC emissions have also been identified when new filament spools are made as this requires thermal melting of the pellets to allow extrusion of the filament [61]. Styrene, ethylbenzene, and benzaldehyde among others were identified from ABS pellets, whilst benzene, styrene, toluene, acetone, methyl methacrylate, butanol, and phenol, among others, were identified from PLA pellets [61]. These VOCs were only identified and not quantified, so the comparison to the extrusion of filament remains qualitative. Recycling filament into new spools was also possible through a two-stage process. When ABS and PLA were recycled into new filaments, 14 VOCs were identified and quantified near and further away from the source. The highest recorded emission was ethanol (87.7 µg/m^3^) from ABS. Following that was methyl methacrylate (57.4 µg/m^3^) and α-pinene (56.4 µg/m^3^) from PLA. Only seven VOCs were above the limit of detection (LOD) during ABS recycling whilst 12 VOCs were quantified during PLA recycling [62]. All concentrations fell within the workplace exposure limits [42], but were of a magnitude similar to the extrusion of non-recycled filaments (Tables 5 and  6).

Table 6 also summarises the difference in the type of emissions when two types of filaments were used. The highest recorded emission rate was styrene, at 6.4 µg/min, from ABS filaments. In Table 6 for PLA filaments, the highest recorded emission rate of 0.25 µg/min was observed for methyl methacrylate. ABS was also observed to emit more VOCs at higher concentrations than PLA. However, the majority of research has focused on emission mass per volume of air, rather than per unit of time, resulting in the same filaments in higher values per cubic meter and VOCs (Table 6).

For ABS filaments styrene concentrations ranging from 2.0 to 2216 µg/m^3^ and for PLA concentrations of methyl methacrylate up to 1.7 µg/m^3^ were recorded. The styrene emissions of 2216 µg/m^3^ were the highest recorded emissions (Tables 5 and  6). Ethylbenzene was the next highest emission identified and ranged from 368.7–647.3 µg/m^3^, for ABS filaments.

## Discussion

### Previous studies

Research on 3D printers has focused on laboratory-based testing, using exposure chambers, and simulated ‘real world’ settings. There seems to be a knowledge gap about the use of desktop 3D printers in home settings and the potential for user exposure [63]. In addition, each of the previous research studies has considered slightly different testing scenarios, both in terms of the environment and the sampling protocol, making comparisons between studies more difficult. A method of standardisation should be adopted for testing in different environments to allow comparison and validation of research. For example, a chamber of a consistent size and ventilation or a room environment with a consistent air exchange. The sensors and samplers used should also be arranged in a consistent placement to the source to allow for comparisons between studies.

During their research, Stefaniak et al. [51] stated that it is unknown whether the VOCs or the ultrafine PM, or a combination of the two, were mainly responsible for any negative health effects or biological changes occurring post-exposure. This shows that further toxicological studies should be carried out to assess the impacts of PM and the VOCs identified from 3D printing. These toxicological studies may include cell death in response to exposure, proteomic response to exposures or DNA/RNA changes, based on exposures to the mixtures of VOCs emitted from 3D printers in addition to previous studies looking at particles emitted. These would offer insight into the short-, and long-term responses of the body to the effects of these chemical exposures.

Whilst each of the identified VOCs was well below the occupational limit values, the combined effects of printer VOCs have not been considered i.e., the TVOC concentration. In addition, the mixture of VOCs may interact with each other or the environment forming secondary VOCs/particles not primarily emitted from the printing process. The presence of these secondary VOCs may further increase TVOC. Owing to the large variety of chemical structures identified from past research, the combination of VOCs may also have the potential to interact with or exacerbate health implications.

#### VP printing

During the previous research conducted by Väisänen et al. [32], the composition of the identified VOCs differed depending on the resin used. The alcohol compounds identified during dental printing were not present during clear resin printing, and the methacrylate compounds identified from clear resin printing were not among the most abundant during dental resin printing. This difference in VOC emissions may relate to the different compositions of these two resins formulated to provide different properties for the printed component. The different molecules used in the resin liquid would interact causing the bond strength to differ as well as the flexibility of the overall structure. Smaller molecules which can bind together more strongly may create a more rigid structure, whilst longer molecules may introduce flexibility into the item e.g. Polyurethane [10]. In addition to the physical properties, the overall health impact would be dependent on the purpose of the item being created. Dental or medical resins which would have direct, long-term contact with the body would also need to be assessed for the potential for harm at a more rigorous level as observed by fewer TVOC emissions by Pham [64], or by specifically looking at biocompatibility and the ability of the object to survive sterilisation [65], which may impact the chemical composition further.

Research conducted by Pham et al. indicated that biological-based resins are adapted for their purpose and as such emit fewer VOCs than their non-biological counterparts [64]. Tough resin was found to emit ten-fold greater emissions than BioMed or Surgical resins after the post-processing curing process [64].

During research conducted by Väisänen et al., the post-processing stage of the resin print procedure was reported to have a twenty-fold increase in the amount of VOC emissions recorded than the active printing stage. This twenty-fold increase in emissions during the post-processing stage of resin printing may indicate that further emission mitigation techniques may be required for this stage when the operator is more likely to be handling the printed component [32]. This was supported by Yang et al. [49] who discussed the phenomenon of VOC emissions becoming trapped within printer hoods, only to be released in much higher concentrations when the operator opens the hood to remove the printed structure. The increase in VOC emission during the post-processing is likely due to the washing of the printed structure in alcohol before over-curing under UV light. The VOC increase can be attributed to the alcohol bath, in addition to the opening of the UV hood surrounding resin printers, releasing any VOCs trapped within the hood into the greater environment.

When the printing process was investigated by Bowers et al. [38] the post-printing tasks including IPA washing and curing were also found to increase VOC emissions. The authors also established that pre-printing processes, including pouring the resin into the resin bed, emit high concentrations of VOCs and are a further potential exposure for the operators, despite being the task with the shortest duration. Within the entire printing lifecycle, each process emitted a different mixture and quantity of these compounds. IPA was prominent during the cleaning stages, whilst 2-hydroxypropyl methacrylate was quantified up to 58.5 µg/m^3^ during the recovery stage after printing ended. Acetaldehyde, acetone, ethanol, and styrene were also quantified at their highest concentrations in the recovery stage. This leads to the potential operator exposures being greater after the printing ends, rather than peaking during the printing process.

#### ME printing

The methods used for exposure and sample collection varied between studies. The majority of exposure measurements were taken inside exposure chambers, which varied in size from 0.18 m^3^ to 3 m^3^ [7, 22, 26, 34], and exposure sampling rooms, 81 m^3^ to 126 m^3^ [7, 24], whilst the samplers themselves were in different positions within their environments. The difference in these environments and sampling protocols complicates direct comparisons. In addition, the measurements were reported using different units. Therefore, the reported values have been converted into standard units to be more easily comparable (µg/m^3^ or µg/min).

Previous research discussed whether if the printer had been used previously that day, this would increase emissions [66]. The use of 3D printers after being in an inactive state would be more representative of hobbyists and home users, rather than industrial use where the 3D printer may be continuously used.

For each filament type (ABS and PLA), concentrations from VOC emissions are discussed in Table 5 and emission rates are tabulated in Table 6. The studies provided consistent evidence for the same types of emissions when the same filament was tested. The identity and concentrations of the VOCs are mostly consistent between the studies, strengthening the overall findings. However, for ABS filament there were ~200-fold differences in the concentrations of acetaldehyde and ethanol emissions. The differences in acetaldehyde and ethanol emissions found between studies could be due to the testing methodology being different as well as the sample collection and sample analysis methods. The differences in collection and analysis methods may account for minor changes in the VOC concentrations quantified, however, the greatest differences are expected to be the test methodology, including print time, distance from the printer and the room volume.

For the VOCs identified from previous literature, the printer emissions fell below the recommended time-weighted average exposure limits in their safety data sheets [42], indicating a limited risk for operators of a single ME printer over 8 h. The TVOC, any repeated exposures, and pre-existing risk factors should be considered for long-term implications to health.

### Printing variables that affect VOC emissions

#### VP printers

Published studies have reported that VOC emissions throughout the printing lifecycle are affected by the different printing variables. When cured and uncured printed clear resins were used to build surgical components, curing the product reduced VOC emissions ten-fold [58]. The differences between clear and surgical resins only made a small contribution to these VOC emissions [58], indicating that the cured status of the resin may have a larger impact on VOC emission than the resin type.

The chronology of the resin printing also affected emissions. Peak emissions were identified by Yang [49] after the printing finished and the build plate rose out of the liquid resin vat, leaving a large surface area for volatilisation to occur. Also, an emission peak was observed by Yang [49] when the printed component was post-processed by washing it with alcohol to clean the surface. This is supported by work from Han et al., where TVOC emissions peaked when the build plate rose out of the resin bed [37]. Another study found that user exposure was twenty times greater during the post-processing steps than during the active print cycle [32].

In research led by Bowers, the separate stages of VP printing were investigated. They quantified concentrations of VOCs during pouring resin, printing, recovery, and the curing process [38]. The time spent during the tasks was not correlated with the VOC concentrations, as the pouring stage emitted some of the highest concentrations for all quantified compounds despite being the quickest stage. Additional research led by Zhang identified that a resin printer switched off and cold remained a source of VOC emissions, due to the volatilisation of resin compounds at room temperature [36]. Ventilation and adequate storage were recommended, and exposures should be controlled [36].

#### ME printers

Research has shown that the filament type impacts the quantity and identity of VOCs emitted [29, 35, 67, 68]. Multiple authors found that the colour of the filament also impacts the VOC emissions [31, 35, 41, 49, 67, 69]. However, Zhang et al. [70] found that filament colour was not a contributing factor for ABS emissions, but filament brand and printer brand were both significant variables with *p* < 0.0001 Alternatively, for the PLA filament, Zhang et al. [70] found that none of the factors were significantly important for differences in emissions. The largest differences were seen for the different printer brands.

Increased VOC emissions have been found to occur at higher temperatures, particularly for ABS filament which requires a hotter extruder and printing bed temperature than used for PLA [67]. The number of printer head nozzles did not make a difference to VOC emissions when one nozzle was compared to two nozzles to build a small hair comb [67]. However, the nozzle temperature did make a difference in the VOC emissions [71, 72]. Testing at 200 °C, 230 °C and 300 °C resulted in increases in concentrations of VOC emissions as the temperature increased [71]. The relative humidity was also found to alter VOC emission, with greater emission related to higher humidity [72]. The temperature of the build plate and nozzle were not considered to impact VOC emissions, however, only a small sample size was used in this study and therefore can only indicate a trend [73].

The location of the 3D printer will also affect the VOC concentrations within the environment, depending on the size and ventilation of the room. The ventilation may be increased with open windows, fans, or air conditioning [74]; and may be reduced by closing doors or windows [75].

The total personal exposure is the combination of concentration and duration, which is different from the total emission from the printer. Ventilation rates affect exposure as they can alter the concentration of compounds within the environment, with higher ventilation leading to lower concentrations due to dilution into a greater volume and increased removal of air. The exposure that a person experiences may affect how the compounds impact health.

### Comparison between VP and ME VOC emissions

Many previous studies have focused on filament extrusion printers, due to the stability of the feed material, widescale adoption and affordability compared to other models. Resin bed printers are a more recent development and consequently, fewer studies about emissions from this type of printer have been published. Most of the previous research has focused on particle emissions from resin printers, but the presence of VOC has been examined in some studies [26, 32, 38, 55, 56, 75]. In previous research, only a few oxygenate compounds; methacrylates, acetone, benzaldehyde, butyraldehyde, isopropanol, formaldehyde, hexaldehyde and propionaldehyde [32, 56] were quantified from the printing process. There seems to be a gap in knowledge concerning VOC emissions from resin bed printers, and specifically for home users where ventilation may be poor compared to industrial settings. Personal exposure studies would benefit research into the safety of affordable 3D printing technology, which is becoming more mainstream in daily life for many work and school sectors.

The differences in identity and quantity of VOCs emitted from the two types of filaments used during ME 3D printers are summarised in Tables 5 and  6. The considerable number of VOCs identified from various filaments and past research for ME shows how varied the VOCs are. In comparison, Table 4 lists the VOCs quantified during resin bed printing as mainly carbonyl compounds or methacrylate compounds. This difference in composition is likely to be caused by the composition of the feedstock materials themselves. The VOCs emitted from the thermoplastics mainly derived from the compounds that the filaments were made from. While the resins emit the monomers used to make the polymer. The variety of VOCs emitted can lead to varied exposures and therefore varied implications for the human body.

An additional difference between VP and ME printing is the temperature of the processes. Whilst ME printing melts the thermoplastics at high temperatures, VP printers only increase to around 40 °C. The VOC emission is dependent on temperature for liquids, as increased temperatures allow more energy per molecule and a higher chance of the molecule partitioning into the gas phase and being emitted as VOC emissions.

### Impacts on health from VOCs emitted from 3D printers

Each of the VOCs quantified from previous research was emitted by 3D printers at concentrations well below the published 8-h GB WEL values, noting the caveat that the printer studies relate to emissions and not personal exposure assessments to VOCs. It is unlikely that the operators of the 3D printers would have continual exposure for eight hours at the same emission rate, due to moving within rooms, leaving rooms, increasing ventilation, and shorter print times amongst other reasons. Work carried out by Runstrom et al. identified the amount of time that VP and ME 3D industry printer operators spent on non-3D printing tasks was 97% and 96% respectively, limiting potential exposure periods [76]. Owing to the probability of a shorter or lower exposure, the dose that the user is exposed to is likely to be even lower than the GB WELs for these VOCs.

One of the more commonly identified VOCs from ME and VP printers is methyl methacrylate [22, 25]. Methyl methacrylate has been identified as a respiratory irritant [77] but its status as a respiratory allergen is uncertain. Methyl methacrylate caused lung inflammation in mice after they were exposed to 150 ppmv (6 × 10^5 ^µg/m^3^) for 120–200 min [78]. However, when Muttray et al. [79] exposed volunteers to 50 ppmV methyl methacrylate (2 × 10^5 ^µg/m^3^) over four hours in a test chamber they found no significant impact on the exposed volunteers other than reports of headaches, which diminished 35 min after exposure [79]. Overall, a review assessing methyl methacrylate determined there was insufficient evidence to classify it as a respiratory sensitiser [80]. The GB WELs are 50 and 100 ppmV for the 8-h time-weighted average and short-term exposure limits respectively [81]; and are several orders of magnitude higher than the concentrations of VOCs quantified from printer emissions [22, 23, 25, 32].

A similar compound linked to 3D printing is 2-hydroxypropyl methacrylate [32]. This compound has been linked to skin irritation, eye damage, skin sensitization, and single-exposure inhalation organ toxicity [82]. Though no GB WEL has been set for hydroxypropyl methacrylate, the 8-h time-weighted GB WEL exposure for methacrylic acid is listed as 70,000 µg/m^3^, which is four orders of magnitude greater than the reported printer emissions of 6–8 µg/m^3^ [32]. There are no GB WEL limits for 2-hydroxypropyl methacrylate, however, there is a GB WEL for the structurally and chemically similar molecule 2-hydroxypropyl acrylate which is 2.7 mg/m^3^ [42], which is again, several orders of magnitude greater than identified. 2-hydroxypropyl acrylate is very similar to 2-hydroxypropyl methacrylate in terms of chemical structure, reactivity, size and polarity, and has similar health impacts such as skin and respiratory irritation and sensitization, however, 2-hydroxypropyl acrylate is also a skin corrosive [83].

Benzene and toluene were also reported in 3D printer emissions and have separately been linked with risk for asthma and other respiratory diseases [84]. Benzene is associated with increasing the risk of leukaemia [84] and is considered a multi-organ carcinogen [85] where exposure can cause chromosomal aberrations [85]. From Table 5, benzene was quantified up to 11.5 µg/m^3^. The GB WEL for benzene is 3.25 mg/m^3^ [42], which is 2 orders of magnitude greater than identified.

Toluene has been shown to have neurotoxic effects post-exposure of up to 200 ppm (7.5 × 10^5 ^µg/m^3^) and has an 8-h GB WEL of 50 ppm (1.8 × 10^5 ^µg/m^3^) [63]. These are several orders of magnitude above the literature emission values of up to 61 µg/m^3^. The GB WEL for 8 h is 191 mg/m^3^ and the 15-min exposure with 384 mg/m^3^ [42].

Another major VOC linked with 3D printing is styrene [7, 22, 25, 41, 86]. Ambient styrene concentration was associated with blood styrene concentration [87], as well as being associated with a reduction in the vibrotactile sensitivity of the participants [87] and reduced stability whilst standing on one leg [87]. Styrene exposure has also been associated with poor colour vision [88]. The reported concentrations of styrene from desktop printer emissions are below the workplace exposure limits of 430 mg/m^3^ [42] by several orders of magnitude

Two of the compounds exceeded the UKHSA guidelines for exposures during ME printing, styrene during ABS printing, and formaldehyde during PLA printing. The guideline air quality value for styrene is averaged over a year, so the potential health risks for people exposed to 3D printer fumes are still likely to be low. However, the guideline for formaldehyde was averaged over 30 min, which may potentially place the operator at risk should they exceed a 30-min exposure at the same concentration. As only one out of two PLA filaments exceeded the UKHSA guidelines, the variation between filaments may be high, even when the same material is used. The variation in VOC emission may lead to differing exposures experienced by the operators, and so caution should be undertaken.

The setting of occupational exposure limits and control guidance values do not typically consider vulnerable non-occupational groups. For example, those with pre-existing respiratory conditions such as asthma or COPD [89], or the elderly or young children. Any of these vulnerable groups may experience a non-proportional response to the VOC exposure. Previous research has highlighted associations between exposure to VOCs and symptoms affecting the respiratory, cardiovascular, and neurological systems [89]. In addition to pre-existing risk factors, chronic exposure needs to be considered. The impact of repeated exposure may cause long-term symptoms, even at low doses.

Previous research led by Karwasz investigated the printing habits of ME printer users and modelled potential exposure scenarios when using a non-ventilated hood. 15% of participants reported headaches when using the printer, 70% used printers with an exposed print chamber and 57% did not use any filtration with the remaining participants being unsure [90]. When the modelling scenarios were analysed, the opening of the chamber door resulted in high levels of pollutants within 3 s, regardless of the ventilation within the room [90]. These scenarios identify the possibility of short-term high exposures to VOC emissions from 3D printers.

## Conclusions

The emissions from ME filament printers have been more widely researched as they are popular 3D printers in terms of cost and ease of use. There has been limited research quantifying VOC emissions from resin bed printers, with only four papers that quantified a specific range of organic VOCs, presenting a knowledge gap for further analysis. There were also only nine papers included for the quantified VOC emission from ABS and PLA filaments for the material extrusion 3D printers, identified from the previous literature up until April 2024. These papers were also broader in the range of VOCs quantified from the 3D printing process, with a larger range of VOCs identified. The concentration of these VOCs was almost all under the UK regulatory exposure limits and likely to present a low risk to human health. However long-term exposure and additional risk factors need to be considered.

The HSE occupational exposure limits referenced in this research may be different to other international standards, as each country sets their limits, Therefore, these results may not be applicable considering individual country limits. However, the ALARP practise should still be used when VOC exposures are considered, as reducing the VOC exposure that the 3D printer operators are exposed to will reduce their potential for long term health impacts. This is true when the exposures are still within the current guidelines or limit values set by the governing bodies as limit values are assessed periodically and can be changed with new and emerging evidence.

The health implications of exposure to VOCs from 3D printers are not fully established. Toxicological studies of individual VOCs have shown adverse effects on human health [42, 63, 78, 84, 85, 87, 88] at higher doses of exposure than observed in the emissions from 3D printers. The GB WELs [42] are several orders of magnitude higher than the levels of VOC emissions from desktop 3D printers, suggesting that the health risks are low. However, interactions between VOCs and secondary aerosols as well as TVOC should also be considered, as these may provoke health effects, particularly in vulnerable groups. TVOC is a cumulative measure of all VOCs in a sample of air. The health effects of exposure to TVOC may not equate to the impact of individual VOCs in this mixture. Most of the emissions data has been obtained in laboratory test chamber studies and there is a need for studies of personal exposure in relevant environments (e.g., homes, small offices). Additional studies focusing on changing specific variables such as the temperature of the 3D printer, as well as the conditions of the experimental room including the size, air exchange, temperature, and humidity should be considered as emission-impacting factors. These factors have information included in the methodology of the past literature; however, the room environments have remained consistent through each experimental set.
